# 

**Published:** 1916-10-07

**Authors:** 


Miss Mary Ruck, of Brinkworth, Wilts, who died on June 23, leaving £9,975, gave £100 each to the Victoria Hospital, Bournemouth; the Victoria Hospital, Swindon; and the Cottage Hospital, Malmesbury; and one-half of the residue of her estate to the Royal Agricultural Benevolent Institution.

### Passing Events and Topics.

### St. Thomas's Hospital Medical School.

Entrance Science Scholarships for 1916 have been gained by M. D. Cadman for £150 and R. G. B. Marsh £60, and J. G. Churcher has won the Arts Scholarship of £15 15s.

### Women and Drink.

The Women's Total Abstinence Union, of 4 Ludgate Hill, E.C., has published an attractive poster calling attention to the dangers of alcohol to pregnant and nursing mothers. A copy can be obtained for 2½d. post free, or twelve copies for 1s. 10d.

### Reduction in Panel Doctors' Fees.

The last report of the London Insurance Committee foreshadows that it will be necessary to curtail the payment to panel practitioners for the December quarter. It is proposed that the payment for each patient for the quarter shall be one penny less.

### Mildmay Hospital, Bethnal Green.

This hospital has had to be closed in order to reconstruct the entire drainage system. The last patients were sent out early in July, but it is hoped to reopen the hospital early in December. Though a small hospital of only fifty beds, it occupies a high position in regard to the thoroughness of its training, which is organised in view of the fact that most of the nurses intend to work as missionaries in places remote from civilisation.

### More Rewards for Gallantry.

Since the publication of the Supplement to the *London Gazette*, referred to in The Hospital last week (page iv), a further Supplement has been issued, containing the names of thirteen medical officers on whom honours have been conferred. Temporary Captain N. W. Broughton receives the D.S.O., as also does Temporary Captain D. W. Hunter. The Military Cross has been conferred on the following officers: Captain W. B. Allen, Temporary Captain F. Dallimore, Temporary Lieutenant P. MacD. Little, Temporary Captain J. McI. Morgan, Temporary Captain B. N. Norman, Temporary Captain J. M. Stenhouse, Captain P. Thornton, Captain G. Torrance, Captain W. S. Wallace, Temporary Captain C. McM. Wilson, Captain J. W. Scott. The names of Captain D. W. Hunter and Captain J. W. Scott have appeared in previous lists.

### A Hospital Ship for the Pacific Islands.

The Rockefeller Foundation, it is stated by the *Lancet*, is providing a hospital ship for service in the Sulu Archipelago, a small group of islands lying south of the Philippines, inhabited by the Moros and near-related tribes. There are about 200,000 of these people, who follow the Mohammedan faith and lead a nomadic or semi-nomadic life. They are descendants of the famous Malay pirates that terrorised the Malay Seas and devastated the Philippine Islands to the north. For more than 200 years efforts have been made, principally through the use of military force, to bring these tribes under the influence of civilisation. Spain was unable to accomplish this, and similar efforts made by the United States have not met with much greater success. Preliminary investigation shows that the medical needs of the people are great. The establishment of dispensaries at different places in the Sulu Archipelago similar to those in the more northern islands is not practical, because they would only reach an inconsiderable fraction of the inhabitants, who are scattered over a large number of small islands.

### Gifts to Hospitals.

MISS A. BECKETT DENISON, of Doncaster, has left £500 to Doncaster Infirmary and Dispensary.

THE London Hospital has received £1,000 in 4½ per cent. Treasury bonds from Sir William Hartley, and £8,000 in response to Lord Knutsford's appeal.

MR. JOHN ELLIS, of Leicester, who died on February 29 last, left £100 to Leicester Infirmary and £100 to the Leicester Institute for the Welfare of the Blind.

MR. G. S. CLARK, of Bristol, who died on June 23, left £1,500 to the Longmore Hospital for Incurables, Edinburgh, and £100 to the Royal Infirmary, Bristol.

MR. G. W. CORBET, of Wellington (Salop), left £50 to the Salop Infirmary and £50 each to Wellington Cottage Hospital and Dispensary, payable one year after the declaration of peace.

MR. LAWRENCE COTTON, J.P., and his wife have given £500 to name a cot at the Royal Infirmary, Blackburn, in memory of their son, Lieutenant John Cotton, who was killed in France on July 1. Mr. Clement Cotton and his wife have given a like sum to name a cot in memory of their son Arthur, who was killed in action at the Dardanelles on June 25, 1915.

### WHAT'S WHAT COLUMN.

[*To meet the wants and wishes of many readers we have organised this Column. Its usefulness must depend upon its continuous use by Matrons, Sisters, and hospital workers.*]

### ANSWERS TO CORRESPONDENTS.

**Valuation of Gifts in Kind.**

TREASURER.—We have religiously valued all gifts in kind, such as those received from the Colonial Governments, and expressed them as directed by King Edward's Hospital Fund on each side of the income and expenditure account. Yet our hospital shows larger receipts in this respect than others of similar size, although we have reason to believe that we have not been unusually favoured in the matter of gifts. Is there any variation in the method of valuation? We do not want unduly to increase the figure representing our cost per occupied bed.

*** Perhaps the hospital has in actual fact come off well in the matter of gifts. The Red Book and supplementary circular of June 1915 make it clear that a hospital need show only a figure representing what has been saved by a gift. Presents of game, fruit, etc., need not be valued. The prices in current contracts and not the market price of the day is the basis of valuation. There should be no variation in method, but this is one of those cases where individual judgment comes into use, and another instance where a general auditor for London hospitals would ensure uniform accountancy, as advocated in an article in THE HOSPITAL of February 12, 1916.

[NOTE.—*In answer to inquiries it is stated that questions of general interest only are dealt with in this column.*]

### Matrons' and Sisters' Appointments.

BELFAST, ULSTER VOLUNTEER FORCE HOSPITAL.—Miss May E. Johnston has been appointed matron. She was trained at St. Thomas's Hospital, and has been staff nurse at the Ulster Volunteer Force Hospital.

CHICHESTER. ROYAL WEST SUSSEX HOSPITAL.—Miss Parsons has been appointed matron. She was trained at the London Hospital, where she has since been sister. She has also been matron of Jessop Hospital, Sheffield.

LEWIS HOSPITAL, STORNOWAY.—Miss Margaret W. Galbraith has been appointed matron. She was trained at Dumfries and Galloway Royal Infirmary, and has since been assistant matron of Bedford County Hospital.

STIRLING COMBINATION HOSPITAL.—Miss A. W. Winrain has been appointed matron. She was trained at Edinburgh City Hospital, and has since been sister at Stirling Combination Hospital, and matron of the Clark Hospital, Largs, Ayrshire.

MITCHAM, HOLBORN MILITARY HOSPITAL.—Miss F. E. Fenn has been appointed sister. She was trained at St. Marylebone Infirmary, and has been holiday sister at Charing Cross Hospital.

ROYAL SEA BATHING INFIRMARY, MARGATE.—Miss M. Wilson has been appointed ward sister. She was trained at the Royal Sea Bathing Hospital, Margate, and was for three years at the York County Hospital, where she has been staff nurse.

**NOTE.—Particulars of Appointments for insertion in this column will be welcomed.**

RATES OF SUBSCRIPTION: Twelve months. 6/6; Six months, 4/-; Three months, 2/-; post free to any address in the United Kingdom. Abroad, Twelve months, 8/8; Six months, 5/-; Three months, 2/6, post free. THE HOSPITAL "Index" to Volume LX., April to September 1916, is now ready, price 1½d. per copy, post free. Address The Manager, "THE HOSPITAL," The Hospital Building, 28/29 Southampton Street, Strand, London, W.C.

